# Usnic acid deteriorates acidogenicity, acidurance and glucose metabolism of *Streptococcus mutans* through downregulation of two-component signal transduction systems

**DOI:** 10.1038/s41598-020-80338-6

**Published:** 2021-01-14

**Authors:** Arumugam Priya, Chandra Bose Manish Kumar, Alaguvel Valliammai, Anthonymuthu Selvaraj, Shunmugiah Karutha Pandian

**Affiliations:** Department of Biotechnology, Alagappa University, Science Campus, Karaikudi, Tamil Nadu 630003 India

**Keywords:** Drug discovery, Microbiology

## Abstract

The principal etiological agent of human dental caries, *Streptococcus mutans* is a multi-virulent pathogen that can transform commensal oral microbial community to plaque biofilms. Major virulence factors that are associated with the cariogenicity of *S. mutans* include adhesion, acidogenicity and acidurity. All these pathogenic traits coordinate and alter the dental plaque ecology which provide room for interaction with other similar acidogenic and aciduric bacteria. This cariogenic flora increases the possibility of enamel demineralization which headway to caries development. The present study was aimed at evaluating the antimicrobial and antiinfective potential of a lichen secondary metabolite usnic acid (UA) against *S. mutans.* Minimum inhibitory concentration (MIC), Minimum bactericidal concentration (MBC) and growth kinetics were evaluated to determine the antimicrobial potential of UA against *S. mutans.* UA at 5 µg mL^−1^ and 10 µg mL^−1^ concentration were considered as MIC and MBC respectively. Effect on biofilm formation was microscopically assessed and found to be reduced in a concentration dependent manner. Gene expression of *gtfB, gtfC, gtfD, vicR, ComDE* and *smu0630* was found to be downregulated upon treatment with sub-MIC of UA. Acidogenicity, acidurity, eDNA synthesis and response to oxidative stress were found to be attenuated by the influence of UA. It was also demonstrated to act on preformed mature biofilm of *S. mutans.* Moreover, UA was shown to possess very low frequency to acquire spontaneous resistance development in *S. mutans.* Besides, no morphological aberrations or toxic effect was instigated by UA in the human buccal epithelial cells as well as to the oral commensals. Altogether, these results demonstrate the therapeutic potential of usnic acid in the treatment of *S. mutans* infection.

## Introduction

Dental caries and periodontist are predominant human microbial infectious diseases often allied with high socioeconomic impact^1^. These oral diseases are not restricted to localized infections but possess the prospects enduring major complications due to invasion to neighbouring anatomical sites^2–6^. If left unattended, both the localized infections may transfigure to chronic diseases.

In dental plaque formation, *Streptococcus mutans* has been implicated as the primary etiological agent due to its proficiency to sense and adapt to diverse environmental stress conditions during host colonization and biofilm formation^7^. As *S. mutans* resides exclusively in the oral cavity, its persistent survival in the oral region is significantly posed with major challenges due to counteraction of surplus immune and non-immune host defense systems such as antimicrobial peptides in the saliva, mechanical shear forces, antimicrobial attack, alterations in the nutrient availability, recurrent acidic condition and other environmental circumstances. To endure such complex harsh residential conditions, *S. mutans* have acquired multiple pathways which facilitate them to adhere, outcompete and interact with the microbiota through which they initiate and progress the infection^8^. Two-component Regulatory Systems (TCRS) are such response regulators which assist *S. mutans* to survive through environmental changes^9^. Whole genome of *S. mutans* has been sequenced completely from which existence of fourteen putative TCRSs have been identified^10,11^. One among these TCRSs is the VicRK signal transduction system which regulates various physiological processes and virulence phenotypes of *S. mutans* including the expression of *gtf*BCD, biofilm formation, oxidative stress response, acid tolerance, bacteriocin production, genetic competence and cell death^12–14^. ComDE is another quorum-sensing signalling system that is essential for genetic competence and biofilm formation^15^. These molecular circuits assist *S. mutans* to colonize on the tooth surface thereby altering the symbiotic oral ecosystem through shifting the non-pathogenic commensal flora to plaque microbiota which eventually progress the caries development^16^. Prime factors that contribute to plaque build-up and teeth erosion by *S. mutans* are acidogenicity, the ability to produce various organic acids through carbohydrate fermentation mechanisms; acidurity, proficiency to survive in low pH environment and biofilm formation^17,18^.

The architect of plaque biofilm restricts the penetrance of antimicrobial agents while in due course, planktonic cells develop novel phenotypes and become less sensitive to the antibiotics^19^. In addition to decreased penetrance, biofilm cells can acquire resistance to antibiotics based on other phenomenon viz mutations, presence of drug efflux pumps and production of neutralizing enzymes and prolonged usage leading to modification in the drug targets etc.^20^. Hence biofilm cells are known to evade antibiotics and host defense mechanisms. Moreover, the use of antibiotics and antiseptic agents in the form of mouth washes and other oral care products against oral pathogens has been reported to result in their adverse side effects such as hypersensitivity, secondary infections and teeth staining^21,22^. Furthermore, due to rapid increase in the incidence of antibiotic resistance, it is imperative to identify novel antimicrobial agents with potential to inhibit the various virulence aspects of *S. mutans*.

With this backdrop, the present study investigated the antimicrobial and antiinfective potential of usnic acid (2,6-diacetyl-7,9-dihydroxy-8,9b-dimethyl-1,3(2H,9bH)-dibenzo-furandione), one of the well-known lichen secondary metabolites^23^ for the management of various virulence phenotypes of S. mutans. Usnic acid has been demonstrated to exhibit antiviral^24^, antiprotozoal^25^, antiproliferative^26^, analgesic^27^ and antiinflammatory activity^28^. Also, Usnic acid has been utilized in medical, cosmetic and perfumery applications. This study for the first time illustrates its added therapeutic potential against *S. mutans.*

## Results

### Determination of MIC

**Figure 1:**
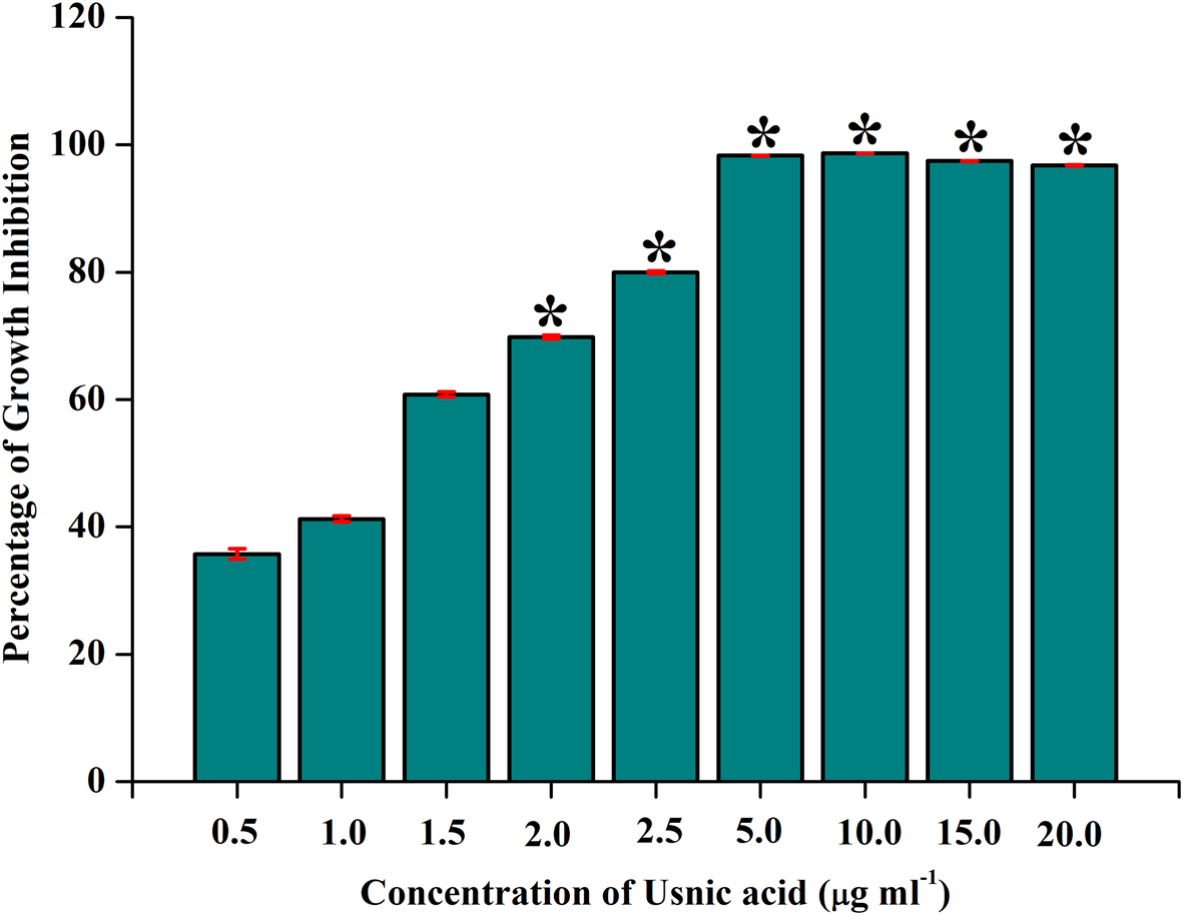
Impact of UA on growth of *S. mutans.* UA significantly inhibited the growth of *S. mutans* in a concentration dependent manner*.* At 5 µg mL^−1^ concentration, visible growth inhibition was observed and the same concentration was considered as MIC. Data are mean ± SD (n = 3) and Asterisk represent significant difference compared with the untreated control (*p* < 0.05).

Initially, impact of UA on growth of *S. mutans* was assessed for lower to higher concentration ranging from 2 to 1024 µg mL^−1^ in which the growth inhibition was found in the least concentration. Subsequently, lower concentration from 0.5 to 20 µg mL^−1^ was evaluated and found that at 5.0 µg mL^−1^ concentration of UA, visible inhibition in the growth of *S. mutans* was observed and hence the same concentration was considered as MIC (Fig. 1).

### Determination of MBC

**Figure 2:**
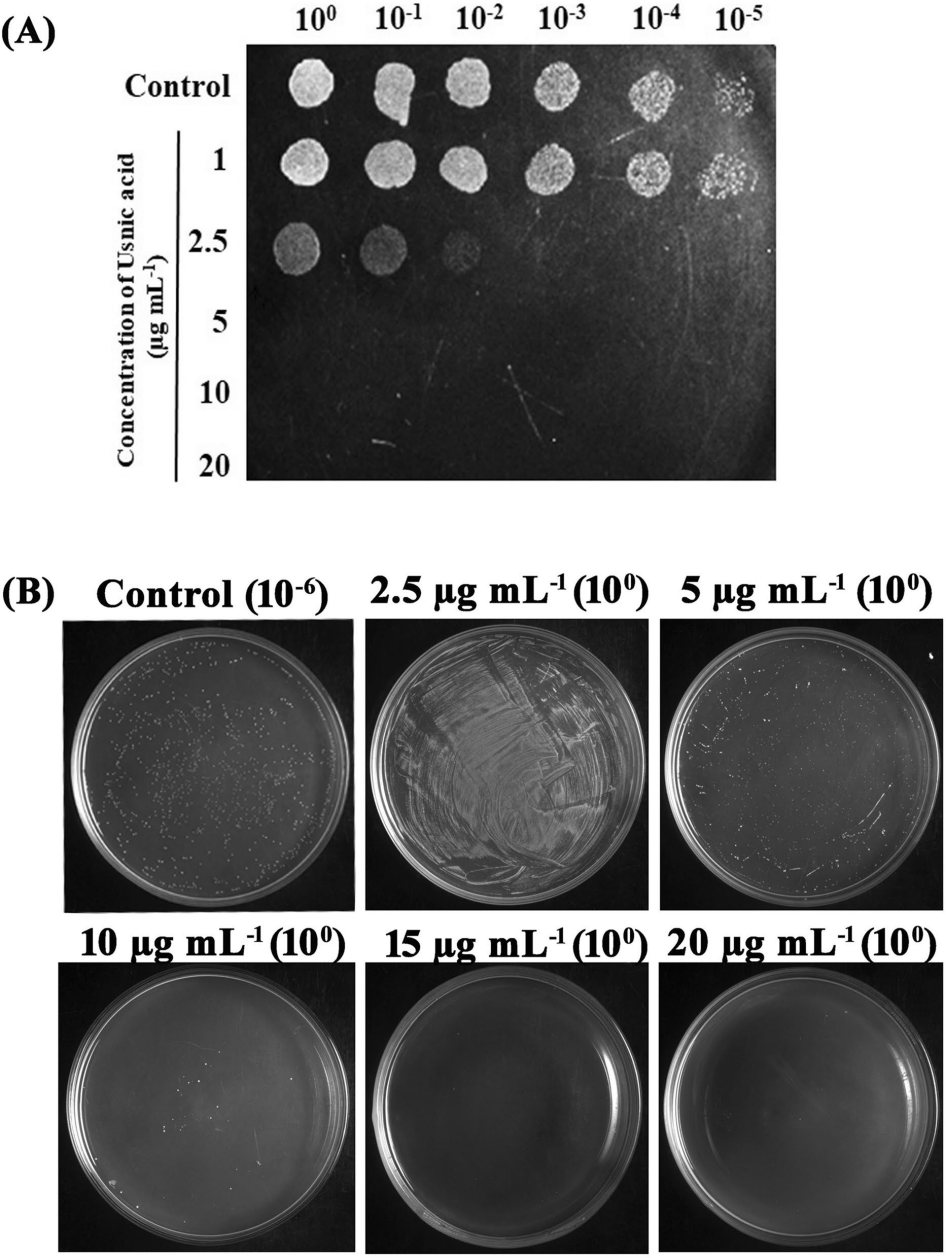
Bactericidal activity of UA on *S. mutans.* **(A)** Spot assay to validate the bactericidal activity of UA **(B)** MBC was determined through cfu assay. From 10 µg mL^−1^, UA was found to be bactericidal against *S. mutans.*

No cells were found at 5 µg mL^-1^ of UA when spotted on THA plates (Fig. 2A). But, viable cells were found at the same concentration when spread plated (Fig. 2B). UA at 10 µg mL^−1^ concentration was found to be bactericidal with less than 5 colonies when spread plated without serial dilution. And no single colony was found in concentrations higher than 10 µg mL^−1^ (15 and 20 µg mL^−1^) (Fig. 2B). Thus, MBC of UA against *S. mutans* was found to be 10 µg mL^−1^.

### Growth kinetics

**Figure 3:**
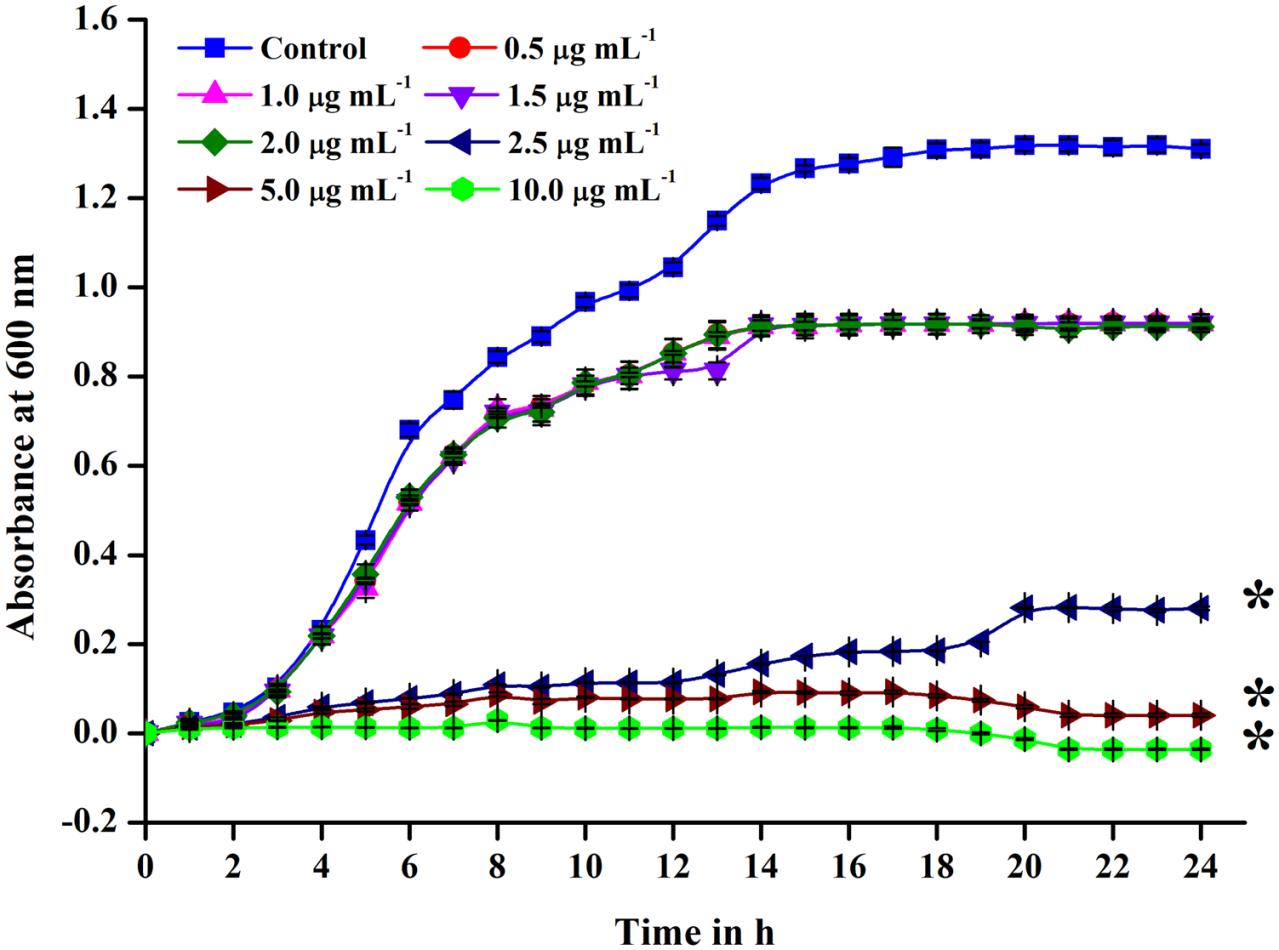
Comparative killing kinetics of UA. MBC, MIC and sub-inhibitory concentrations (0.5, 1, 1.5, 2 and 2.5 µg mL^−1^) of UA was assessed for the time dependent growth inhibition. Substantial reduction in growth was noted when treated with concentration from 2.5 µg mL^−1^ of UA whereas no proliferation of cells was found at MBC from 0 to 24 h.

Proliferation of *S. mutans* in the absence and presence of UA at sub-inhibitory concentrations (0.5–2.5 µg mL^−1^), MIC (5 µg mL^−1^) and MBC (10 µg mL^−1^) was assessed over a 24 h period. Significant inhibition in the proliferation of *S. mutans* was observed at sub-inhibitory concentration starting from around 5th hour of incubation and the proliferation was found to deteriorate drastically afterwards. At MIC and MBC, the proliferation of *S. mutans* cells was constantly supressed throughout the period of evaluation (Fig. 3).

### Effect of UA on biofilm formation

**Figure 4:**
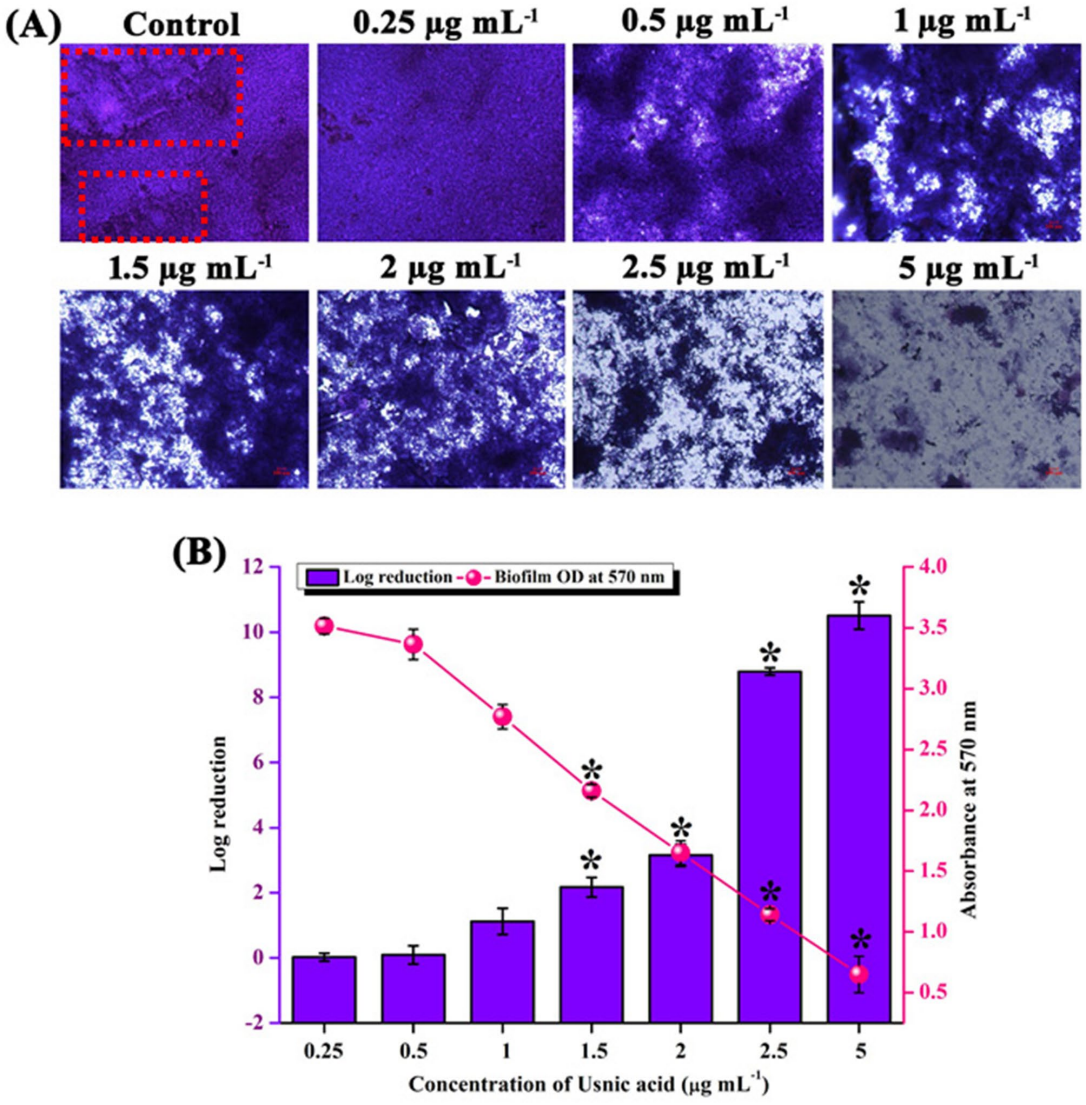
Effect of UA on biofilm formation. (**A**) Dose dependent reduction in the surface attached biofilm formation was observed. Multi-layered biofilm architecture (indicated in red coloured box) in the untreated *S. mutans* sessile life form was found to be absent in the sessile cells of UA treated *S. mutans.* Scale bar indicates 100 µm. (**B**) Biofilm biomass quantified through spectrophotometric method and cfu analysis displaying the concentration dependent reduction in the surface attached *S. mutans* cells under the influence of UA treatment.

*S. mutans,* the robust biofilm former was found to produce multi layered biofilm (indicated in red colored box in Fig. 4) in the absence of UA. Under the influence of UA, even at lowest concentration i.e. 0.25 µg mL^−1^, dearth in the multi-layer form was noted. Though 5 µg mL^−1^ was MIC and the significant growth inhibition was found only above 2.5 µg mL^−1^ of UA, considerable and concentration dependent reduction in the surface attachment of *S. mutans* was observed at non-lethal concentrations. From this, it is evident that even at lower concentration UA could minimally disperse the *S. mutans* cells from adherence.

Similar result was evidenced in the biofilm biomass quantification enumerated through spectrophotometric absorbance and CFU analysis. UA treatment effectively reduced the viable cell count in the biofilm of *S. mutans* from 0.25 µg mL^−1^ of UA. More than 1 log reduction was observed at concentrations from 1 µg mL^−1^.

### Gene expression profiling through real time PCR

**Figure 5:**
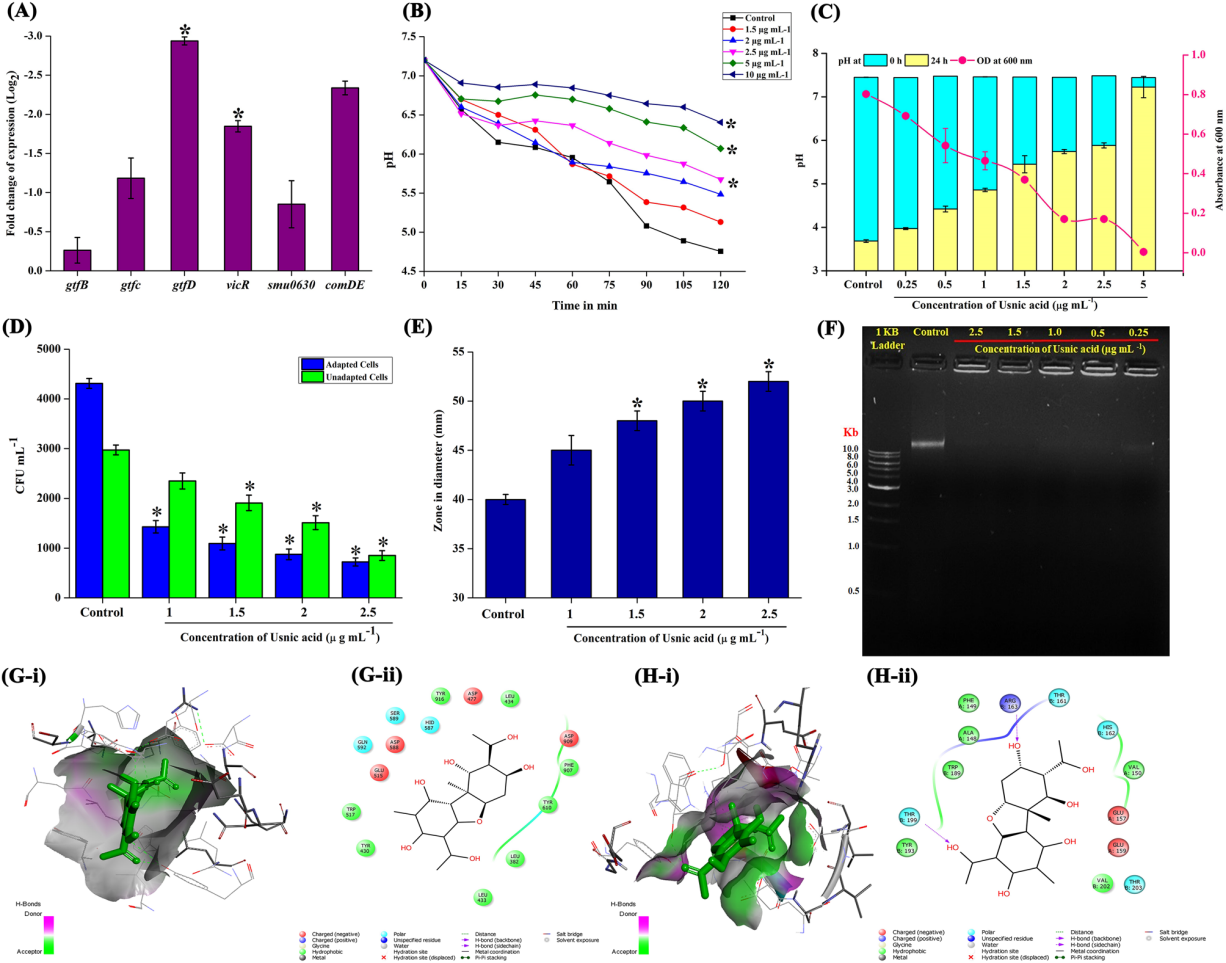
Effect of sub-MIC levels of UA on the gene expression of key virulence and quorum sensing regulators of *S. mutans.* **(A)** Relative variation in the gene expression of *S. mutans* under the influence of 2.5 µg mL^−1^ concentration of UA compared to the untreated control. (**B–Hii)** substantiation of variation in gene expression through physiological assays. **(B) **and** (C)** Effect of UA on acidogenic potential of *S. mutans* which is under the control of ComDE (B-Glycolytic pH drop assay and C-pH of the spent medium)*.* UA at sub-MIC, MIC and MBC significantly inhibited the glycolytic pH drop. Also, acidic nature of the broth was found to be increasing with UA treatment. **(D)** Effect of UA on acid tolerance of *S. mutans* at sub-MIC levels. UA significantly inhibited the survival rate of *S. mutans* cells in acidic condition compared to untreated controls. **(E)** Sensitization of *S. mutans* cells to H_2_O_2_ by the influence of UA treatment. **(F)** Repression of eDNA content in the UA treated biofilm matrix. **(Gi–Hii)** Binding pattern and molecular interaction of UA with *vicR* (Gi and Gii, 3D and 2D respectively) and *gtfC* (Hi and Hii, 3D and 2D respectively)*.* Data are mean ± SD (n = 3) and Asterisk represent significant difference compared with the untreated control (*p* < 0.05).

UA was found to influence the growth and biofilm formation of *S. mutans.* Hence, in order to analyse the gene expression level changes in the regulatory systems that are related to the growth and biofilm, major quorum sensing signal transduction system *vicR,* and other virulence gene such as *gtfB, gtfC, gtfD, smu0630* and *comDE* were determined. Expression level of these genes were found to be downregulated by the effect of UA (Fig. 5A).

### Impact of UA on acidogenicity

*S. mutans* has the ability to ferment wide range of carbohydrates and primarily glucose thereby decreasing pH of the environment acidic. This acidogenic potential of *S. mutans* was reported to be the primary cause of enamel erosion in the tooth which subsequently leads to caries formation. Glycolytic pH drop assay and acidity measurement evidenced that UA holds the potential to limit the acidogenicity of *S. mutans.*

In glycolytic pH drop assay, *S. mutans* in the absence of UA was found to rapidly utilize the glucose through glycolytic pathway which resulted in gradual decrease in the pH from 7.2 to 4.2 over 120 min. On the contrary, UA treatment restricted the rapid fermentation of glucose and at 10 µg mL^−1^_,_ pH was found to be close to neutral (Fig. 5B).

#### pH of the spent medium

In another set of experiment, where the *S. mutans* cells were grown under the influence of UA, initial pH and pH after 24 h of incubation was measured. pH of the medium was found to be reduced in a concentration dependent manner (Fig. 5C).

### Effect of UA on acidurity

Another potential survival ability of *S. mutans* amidst the plaque environment is acidurity. Acid tolerance ability of *S. mutans* under the influence of UA was assessed under two conditions viz. adapted and unadapted cells. Adapted cells are pre-exposed to non-lethal acidic pH for habituation of *S. mutans* cells to acidic conditions before being subjected to lethal pH. Whereas unadapted cells are directly cultivated in lethal pH. From the results, it is apparent that adapted cells exhibited increased survivability in lethal pH than the unadapted cells. Moreover, regardless of the prior acclimatization to acidic condition, UA treatment subsided the viability of *S. mutans* cells under acidic condition when compared to control (Fig. 5D). This result substantiates that UA treatment has the potential to even act on *S. mutans* cells that are habituated to survive under acidic condition (Fig. 5D).

### H_2_O_2_ sensitivity assay

H_2_O_2_ sensitivity assay results revealed that the UA treated *S. mutans* cells were found to be more sensitive to H_2_O_2_ treatment than the control. Zone of clearance of 40 mm in diameter was observed for *S. mutans* control whereas 45, 48, 50 and 52 mm was observed for 1.0, 1.5, 2.0 and 2.5 µg mL^−1^ treated *S. mutans* cells (Fig. 5E).

Impact of short exposure of *S. mutans* cells to H_2_O_2_ in the absence and presence of UA at 5 and 10 µg mL^−1^ concentration was enumerated through CFU analysis. 1.8 × 10^8^ ± 0.45 cells exposed to 50 mM H_2_O_2_ resulted in 1.4 × 10^8^ ± 0.53 cells in the absence of UA. Complete reduction in the viable cells were observed in UA treated cells evidence that UA sensitises the *S. mutans* cells to H_2_O_2_ treatment. Spot assay showing reduction in the cell count is represented in the supplementary figure S1.

### Influence of UA on eDNA content in *S. mutans* biofilm conglomerate

Influence of UA on eDNA content from the biofilm conglomerate of *S. mutans* was qualitatively evaluated by extraction of eDNA followed by agarose gel electrophoresis. Remarkably, eDNA content was found in a detectable level only in the control biofilm. No visible eDNA band was found even in the *S. mutans* biofilm grown in the presence of least concentration of UA (Fig. 5F). This could be due to the release of eDNA to the external environment (supernatant).

### Molecular interactions of UA with VicR and GtfC

The interaction between VicR-UA and GtfC-UA exhibited a binding energy of − 8.0 kcal/mol and − 7.3 kcal/mol respectively (Table 1). The interaction between VicR-UA was stabilized by hydrogen bond interaction with Arg B:163 and Thr B:199, a pi Anion interaction with Glu A:157 and pi-alkyl interaction with Val B:202, Trp B:189 and Arg B:163 (Fig. 5G i and ii). Interaction between GtfC-UA was stabilized through two conventional hydrogen bond with Tyr D: 430, a pi-alkyl interaction with Trp D:517 and a pi-sigma interaction with Tyr D:916 (Fig. 5H i and ii). Strong binding affinity between UA and target proteins further substantiates the downregulation of expression of *vicR* and *gtfC.*

**Table 1:** Molecular interactions and binding energy identified through molecular docking analysis of UA with *VicR* and *GtfC.*

Receptor	Ligand	No of hydrogen bonds	Key residue(s)/Pi Anion	Binding energy score (kcal/mol)
Response regulator VicR domain	Usnic acid	2	Arg B:163 Thr B: 199 Glu A: 157 (Pi Anion)	− 8.0
GtfC		2	Tyr D: 430	− 7.3

### Spontaneous resistance development

**Figure 6:**
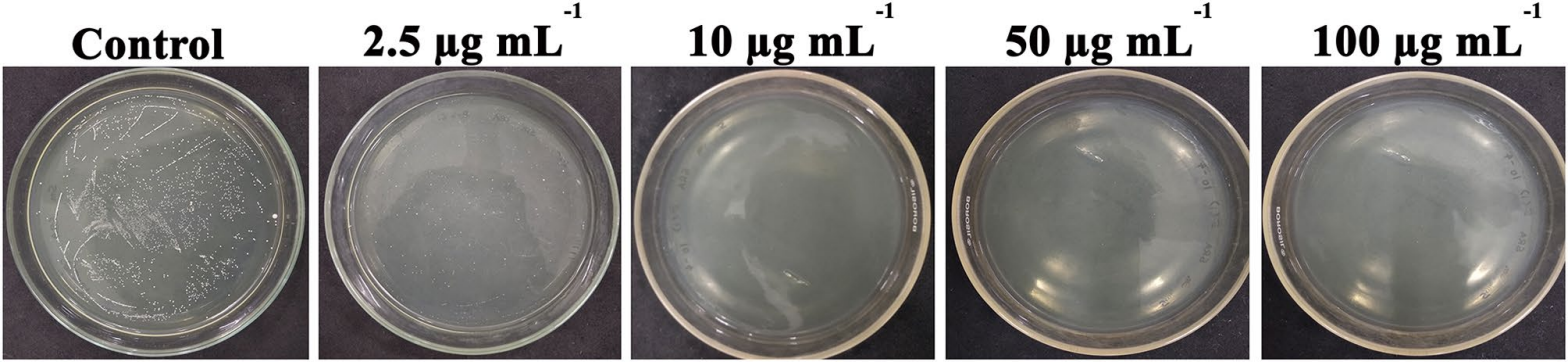
Spontaneous resistance frequency of *S. mutans* to UA. No resistant colonies were zxobserved in the MIC or higher concentration of UA which indicates that UA is a promising agent to treat cariogenic pathogen *S. mutans*.

A common and major concern in the present days antimicrobial therapy is the development of bacterial resistance instinctively. To evaluate if *S. mutans* might develop spontaneous resistance against UA treatment, 10^8^ cells were plated on THA plates containing sub-MIC (2.5 µg mL^−1^) and 1×, 5× and 10× MBCs (10, 50 and 100 µg mL^−1^) of UA. No resistant colonies were observed in the plates except the one added with sub-MIC of UA yet after 72 h of incubation. Accordingly, from the result it is contemplated that UA poses spontaneous resistance frequency of less than 10^8^ (Fig. 6).

### Mature biofilm disruption

**Figure 7:**
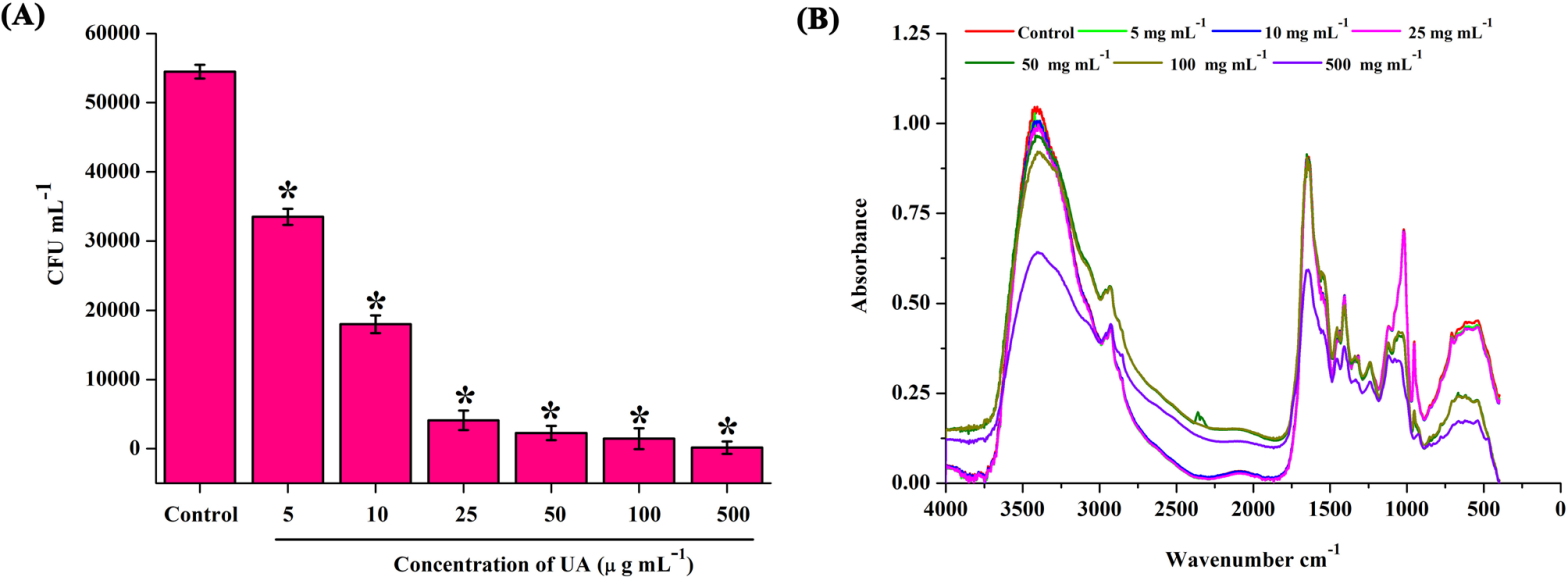
Mature biofilm disruptive potential of UA. *S. mutans* cells in the preformed biofilm mature biofilm was found to be influenced by the UA treatment. **(A)** Significant reduction in the viable cells was observed in a concentration dependent manner. **(B)** Variation in the functional group of the biofilm cells in the absence and presence of UA. Major alterations in the region such as 3800–3000 cm^−1^ and 1900–600 cm^−1^ were observed which corresponds to absorptions of fatty acids and proteins respectively. Data are mean ± SD (n = 3) and Asterisk represent significant difference compared with the untreated control (*p* < 0.05).

Biofilm disruptive ability of UA was assessed through total viability counting by spread plate method. Compared to mature biofilm cells in control, treatment with UA was found to significantly reduce the number of viable cells in preformed biofilm in a concentration dependent manner (Fig. 7A). In addition to this, components of the biofilm conglomerate were analysed through FTIR analysis in the range of 4000–400 cm^−1^. Major alterations were observed in the spectral regions 3800–3000 cm^−1^ and 1900–600 cm^−1^ in all the samples. These regions correspond to the absorptions of fatty acids and proteins respectively (Fig. 7B).

### Assessment of toxicity on human buccal epithelial cells (HBECs)

**Figure 8:**
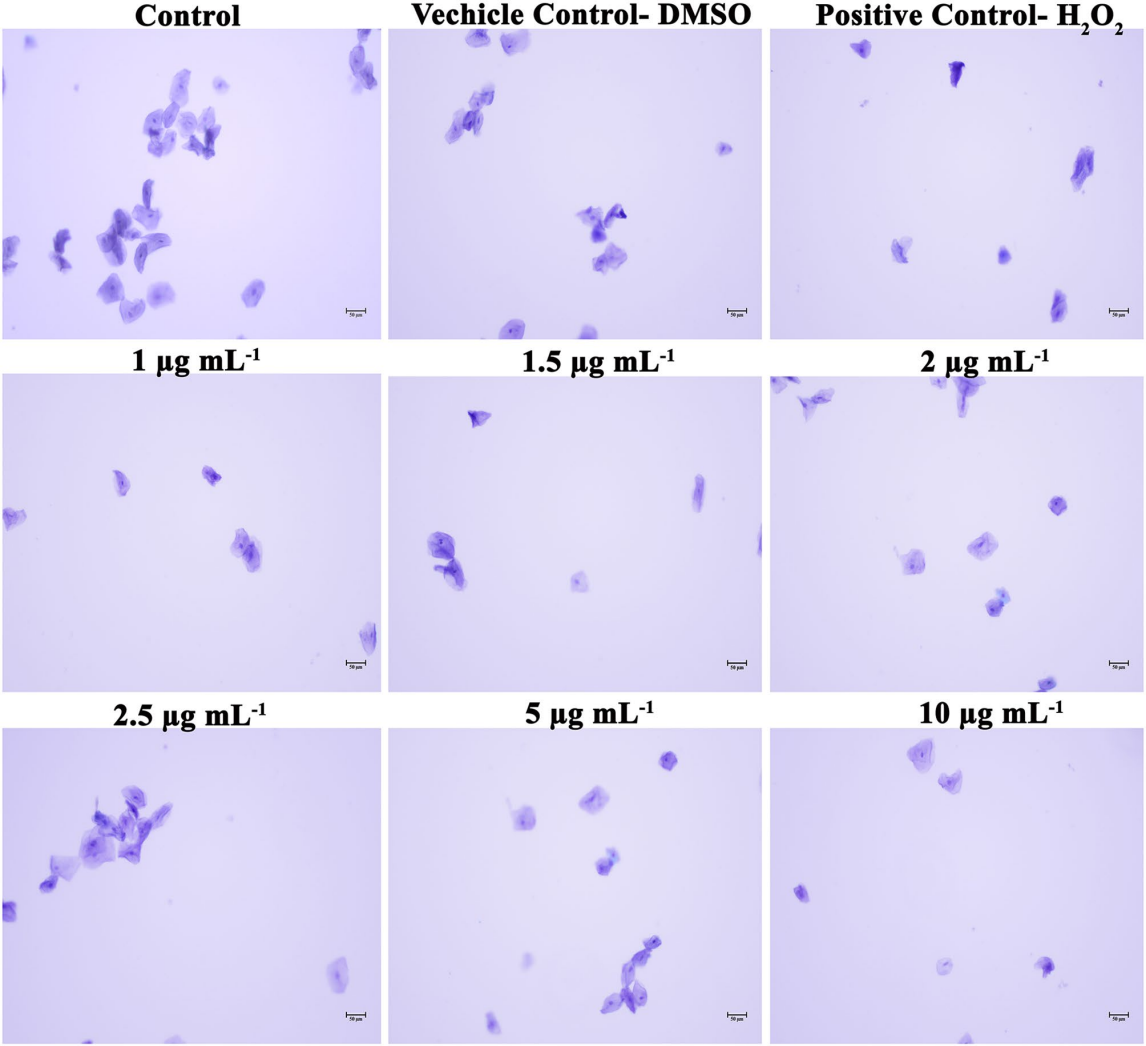
Nontoxic nature of UA to HBECS. HBECs were treated with 1 to 10 µg mL^−1^ of UA and the cells were found to be normal as compared with the control cells with no morphological aberrations. Whereas, H_2_O_2_ treated cells were found to be wrecked and damaged.

HBECs were used to evaluate the safety aspects of using UA in the dentifrice application for the treatment of dental plaque. From the microscopic analysis, it is evident that HBECs were found to be normal and healthy in UA treatment similar to the control. Positive control HBECs which received H_2_O_2_ treatment was found to be have morphological deformities. Hence, this result can be concluded with the note that UA is not lethal to HBECs and can be considered as safe for the oral application (Fig. 8).

### Effect of UA on oral commensals

To extend the therapeutic usage of UA in oral care products, impact of UA on oral commensals was analysed and no significant impact was found when compared to the positive control, chlorhexidine (Supplementary Figure S2). Considering the non-lethal effect of UA on both HBECs as well as oral commensals, the bioactive can be taken for use in dentifrices.

## Discussion

The present study demonstrates the antimicrobial and antiinfective potential of UA, a secondary metabolite of lichen species against *S. mutans* which has been well recognised as a pathogen that initiates the formation of dental caries. UA at MIC, i.e. 5 µg mL^−1^ concentration arrested the growth of bacterium whereas the bactericidal effect was found at 10 µg mL^−1^. MIC and MBC of UA against *S. mutans* was found to be at lower concentration which indicates that minimum concentration of drug is adequate to inhibit the growth of organism. Moreover, the proliferation of *S. mutans* was found to be substantially diminished even at sub-MIC of UA. Drugs with lower MIC values are generally considered as effective antimicrobial agents and thus UA can be regarded as an effective antimicrobial component to treat *S. mutans* infection. As *S. mutans* naturally resides in the human oral cavity and more specifically on the hard surfaces of tooth and in dental plaque, the bacterium often adopts a sessile lifestyle for the survival and insistence in its inherent ecosystem rather than free-living planktonic lifestyle^29–31^. Furthermore, cells that reside in biofilm state exhibit varied phenotypic attributes that are diverse from their planktonic counterparts which is usually accompanied with significant changes in the gene expression pattern^32,33^. Considering the role of biofilm in the virulence attributes of *S. mutans,* effect of UA on biofilm formation and the expression of genes involved in biofilm and its associated factors were analysed. While mentioned earlier, as *S. mutans* predominantly follows a sessile lifestyle it is known to form robust biofilm on dental surfaces^34^. In line with this fact, multi-layered, strong and entire surface covered thick biofilm was observed microscopically in the control *S. mutans* cells. Whereas, the biofilm of *S. mutans* grown in the presence of UA was found to be frail and moderately sparse in a concentration dependent manner. Quantification of biofilm biomass also evidenced that even at lowest concentration, UA could effectively reduce the number of biofilm cells. Influence of UA on biofilm inhibition of *S. mutans* was not due to the growth inhibitory effect as the concentration used to test the effect of UA on biofilm formation of *S. mutans* is analysed from a concentration which is less than 1/16 × MIC, where the growth inhibition was trivial and the biofilm formed was insubstantial. Gene expression analysis of major two-component signal transduction systems such as VicRK and ComDE; Smu0630, a gene which is critical for biofilm formation regardless of the carbohydrate source and extracellular glycosyl transferases (GTFs) such as *gtfB*, *C* and *D* revealed that UA at sub-MIC (2.5 µg mL^−1^) decreased the expression of these genes. *vicR*, a response regulator of the VicRK signal transduction system is reported to be essential for the viability of bacterium and the gene products of *vic* modulate the adherence, biofilm formation and genetic competence development in *S. mutans*^12^*.* In addition, they are demonstrated to regulate the expression of various virulence associated genes that are related with the synthesis of polysaccharides such as *gtfBCD*, *ftf* etc. Downregulation of *vicR* by UA influenced the expression of all three glycosyl transferases *gtfBCD.* Gene products of g*tfB* and g*tfC* results in the synthesis of water-insoluble glucans which are essential adhesive molecules that bridge bacteria to the acquired pellicle around tooth surface^35^. *GtfD* predominantly synthesizes water soluble glucans^36^. Reports have demonstrated that Gtfs are recognized as pivotal contributors for the alteration of biofilm architecture, disruption of homeostasis in the community of healthy microbiota and induction of caries progression^37^. Ren et al., in 2016 have pronounced that molecules that target the glycosyltransferase possess the ability to inhibit biofilm formation and cariogenicity of *S. mutans*^38^*.* Downregulation in the Gtfs B, C and D by the influence of UA demonstrates that the compound can be effectively expended for inhibiting *S. mutans* biofilm and cariogenicity. In addition to this, VicRK system has been demonstrated to respond to oxidative stress. Knockout mutant of *vicK* system has been proven to be sensitive to H_2_O_2_^13^ and similar results were observed in the UA treatment. ComDE is a quorum sensing TCSTS which regulates the formation of biofilm in *S. mutans*^39^*.* This system also regulates the expression of various virulence factors in a cell density dependent manner and affects genetic competence and acid tolerance response^15,40^. UA treatment significantly reduced the expression of ComDE which allude that downstream targets and virulence attributes under the control of ComDE such as biofilm formation and acid tolerance are also influenced which is substantiated by the results of glycolytic pH drop assay, acid measurement and acidurity. Acidogenicity, the ability to produce acid from wide range of fermentable sugars and acidurity, the potential to survive in low acidic conditions are two remarkable capacity of *S. mutans* to initiate caries and progression of teeth erosion^41,42^. In the glycolytic pH drop assay, fermentation of glucose by *S. mutans* was found to be decreased upon UA treatment. From the acidurity results it has been corroborated that UA has the ability to act on survival of *S. mutans* cells that are adapted to survive under acidic condition. Kawari et al., have reported that under low pH conditions, *S. mutans* tend to form biofilm in an eDNA dependent manner^43^. Under Com-dependent biofilm formation at low pH conditions, as a stress response from the lysed cells, induction in eDNA release may be found. eDNA in the biofilm matrix acts as adhesives which further strengthen the biofilm^44,45^. Moreover, upregulation of biofilm formation is also correlated with the presence of eDNA^46^. Also, a mutant study reported that the deletion of vicR showed enhanced release of eDNA in the supernatant^47^. Remarkably, eDNA content in the UA treated biofilm conglomerate was completely repressed when compared to the eDNA from control. This could be due to the enhanced release of eDNA content to the external environment. Furthermore, UA precisely acted on the preformed mature biofilm of *S. mutans.*

**Figure 9:**
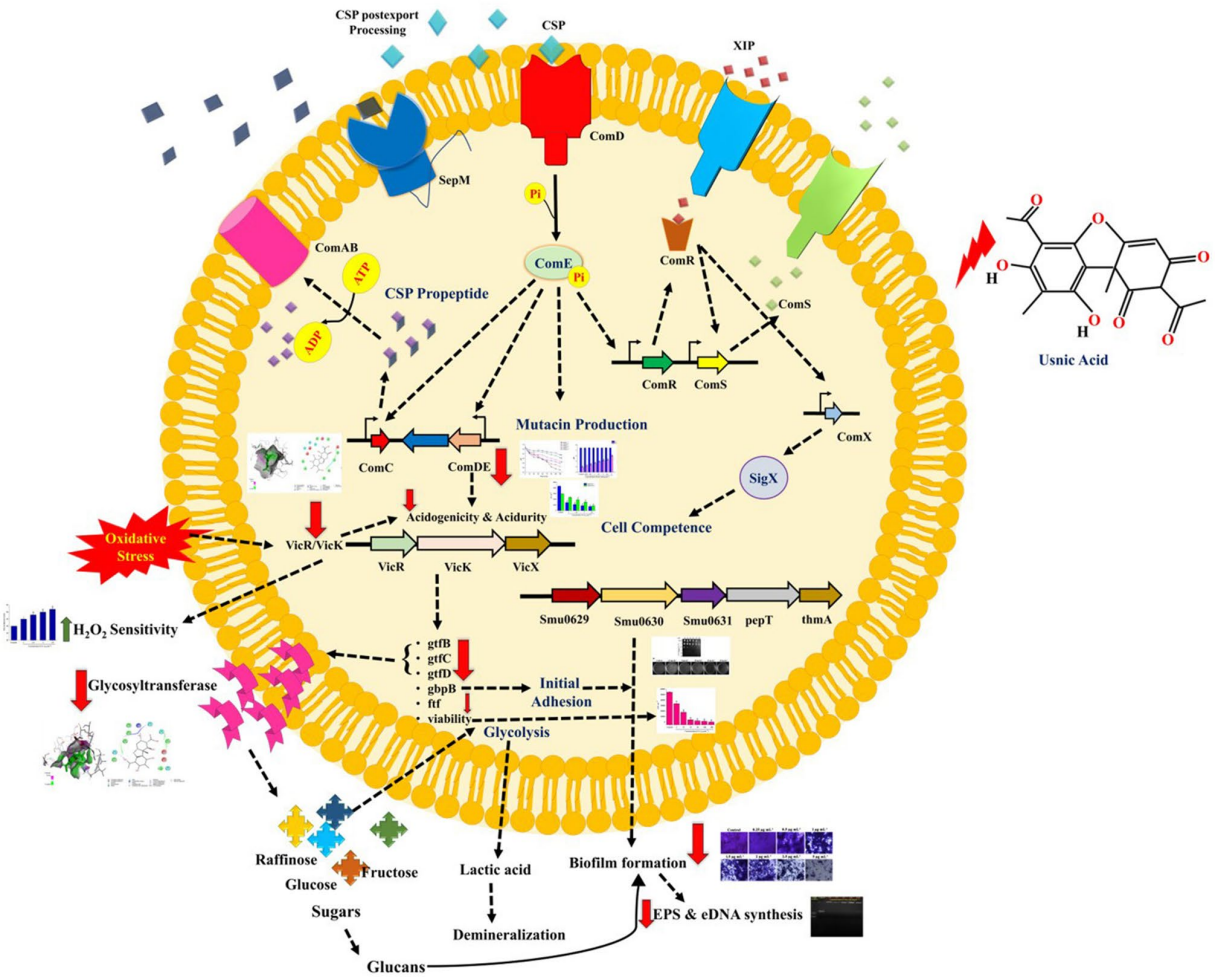
Schematic representation of antivirulence mechanism of UA against *S. mutans.* UA treatment influences the two major TCRS’s VicRK and ComDE which are regulators of major virulence factors such as synthesis of glucosyltransferases, biofilm formation, acidogenicity and acidurity, response to oxidative stress etc. UA also alters the expression of *Smu0630,* which a hypothetical protein postulated to be essential for biofilm formation. Under the influence of UA, the aforementioned virulence attributes of *S. mutans* was found to be attenuated. Red arrow indicates downregulation and green arrow indicates upregulation.

Smu0630 gene was demonstrated to play a role in biofilm formation and it has been suggested as a potential target for novel therapeutics^8^. Downregulation of Smu0630 by UA was observed. Downregulation of key players in the biofilm formation and associated virulence factors by UA imply that probabilities of resistance development by *S. mutans* against this therapeutic is less likely. This has been further substantiated through spontaneous resistance assay where it is found that UA poses spontaneous resistance frequency of less than 10^8^. UA was found to be safe against the HBECs as well as to the oral commensals, which completes its therapeutic potential in treating *S. mutans* infection. Schematic representation depicting the molecular targets of UA in *S. mutans* is portrayed in the Fig. 9.  Altogether, the present study demonstrates the therapeutic antimicrobial and antiinfective potential of UA against persistent dental plaque forming pathogen *S. mutans*.

## Methods

### Ethical statement

Human Buccal Epithelial Cells (HBECs) and saliva sample used in this study for toxicity analysis were collected from the healthy individuals. Written informed consents were obtained. For HBECs individuals were asked to gently rub the mucosal surface of cheeks. Protocol for experimentation and the use of HBECs and saliva were assessed and approved by the Institutional Ethical Committee, Alagappa University, Karaikudi (IEC Ref No: IEC/AU/2018/5). All methods were carried out in accordance with appropriate guidelines and regulations.

### Bacterial strain, culture media and growth conditions

Reference strain *Streptococcus mutans* UA159 from American Type Culture Collection (ATCC 700610) was used in this study. Agar plates containing Todd Hewitt Broth (THB) (Hi-media, Mumbai, India) was used for routine maintenance of the culture. For subculture and performance of bioassays, *S. mutans* was cultured by incubating at static condition for 24 h at 37 °C on THB supplemented with 1% of yeast extract and sucrose (THYES).

### Phytochemical

Usnic acid (PubChem CID: 5646) was procured from Sigma Aldrich, US. Stock solution of 5 mg mL^−1^ of Usnic acid (UA) was prepared in DMSO and stored at room temperature until further use.

### Determination of minimum inhibtiory concentration (MIC)

Growth inhibitory potential of UA against *S. mutans* was determined by microbroth dilution assay in 24 well microtiter plate (MTP) according to the guidelines of Clinical and Laboratory Standards Institute^48^. Briefly, 1% of 24 h grown culture of *S. mutans* was used to inoculate 1 mL of THYES broth. Initial screening was assessed with increasing concentrations of UA ranging from 2 µg mL^−1^ to 1024 µg mL^−1^. Later, inhibitory effect of concentrations ranging between 0.5 µg mL^−1^ to 20 µg mL^−1^ was tested. Appropriate vehicle and negative controls were maintained parallelly. Following 24 h of incubation at 37 °C, visible difference in the growth was observed and the cell density was evaluated by measuring absorbance at 600 nm in multifunctional spectrophotometer (Spectra Max 3, Molecular Devices, USA).

### Determination of minimum bactericidal concentration (MBC)

To ensure the growth inhibitory potential and determine the MBC of UA against *S. mutans*, cfu and spot assay were performed. Briefly, 1% of 24 h grown culture of *S. mutans* was incubated in the absence and presence of various concentrations of UA which was predetermined through MIC assay. Following 24 h of incubation at 37 °C, cells were serially diluted and 5 µL from each dilution was spotted on THA plates and incubated for 24 h at 37 °C. Control (serially diluted) and treated groups (without dilution) were also spread plated on THA plates and incubated at 37 °C for 48 h. Further, the results were documented using Gel documentation system (Bio-Rad Laboratories, XR+, USA).

### Growth kinetics

Cell proliferation of *S. mutans* over 24 h period of time with 1 h interval was assessed to evaluate the effect of UA on growth kinetics of *S. mutans.* Briefly, overnight grown culture of *S. mutans* was used to inoculate 50 mL of THYES broth. UA at various concentrations 0.5 µg mL^−1^ to 10 µg mL^−1^ were supplemented in the test samples whereas control received DMSO (vehicle control) and incubated at 37 °C. At every time point, 200 µL of culture was drawn from each tube for assessing the rate of growth by measuring the optical density (OD) at 600 nm using multifunctional UV–Vis spectrophotometer (Spectra Max 3, Molecular Devices, USA).

### Effect of UA on biofilm formation

#### Microscopic visualization of biofilm architecture under the influence of UA

For microscopic visualization, *S. mutans* biofilm was allowed to form on 1 cm × 1 cm glass surface immersed in 1 mL of THYES broth in the absence and presence of various concentrations of UA (0.25 µg mL^−1^ to 5 µg mL^−1^). Subsequently, at the end of incubation period for 24 h at 37 °C, the glass slide pieces were taken out from the medium, rinsed twice in PBS and allowed to air dry. Later, the slides were stained with 0.4% crystal violet and incubated for 10 min. Surplus unbound stain was removed by a gentle rinse in distilled water and air dried. Biofilm cells stained with crystal violet was then visualized under light microscope (Nikon Eclipse 80i, USA) at a magnification of X400 and the images were documented with the accompanied digital camera.

#### Quantification of biofilm biomass

Number of viable cells in the biofilm of *S. mutans* in the absence and presence of UA was quantitatively analysed through CFU analysis. Briefly, biofilm assay was performed in the absence and presence of various concentrations of UA in 24 well MTP. Following incubation at 37 °C for 24 h, the supernatant was discarded and the biofilm cells were washed with sterile PBS to remove unbound and loosely bound cells. Biofilm cells adhered to the surface was scrapped out and aspirated well in PBS. Cells were serially diluted in PBS and spread plated on THA plates, incubated at 37 °C for 24 h. Number of cells in the biofilms of control and treatment was enumerated^49^. Quantification was also performed through spectrophotometric analysis where the biofilm cells adhered to the surface of 24 well MTP in the absence and presence of UA was stained and destained with 0.4% crystal violet and 10% glacial acetic acid, respectively. Destained solution was read at 570 nm using multifunctional UV–Vis spectrophotometer (Spectra Max 3, Molecular Devices, USA).

### Gene expression profiling through real time PCR

Total RNA was extracted from *S. mutans* cells cultured for 24 h at 37 °C in the absence and presence of UA at sub-MIC concentration by Trizol method of RNA isolation according to the manufacture’s protocol. Isolated RNA was dissolved in 30 µL of 0.1% diethylpyrocarbonate (DEPC)-treated water and the quality check was performed by agarose gel electrophoresis. Subsequently, extracted RNA samples were converted into cDNA using High-capacity cDNA Reverse Transcription Kit (Applied Biosystems, USA) in the thermal cycler (Eppendorf, Germany). cDNA was quantified using Bionano Spectrophotometer (Shimadzu, Kyoto, Japan).

Expression profile of candidate genes were examined through qPCR analysis. List of candidate gens, their function and primer sequences are mentioned in Table 2. Reactions were performed in a total volume of 10 µL. Equal quantity of cDNA from control and UA treatment was used as template. Primers for the specific genes were added individually with the SYBR Green Master Mix (Applied Biosystems, USA) reagents at a predefined ratio and analysed using the thermal cycler (7500 Sequence Detection System). The expression pattern of candidate genes was normalized against 16S rRNA (housekeeping gene) and quantified using the ^ΔΔ^ CT method^50^.

**Table 2:** List of genes, their influence in virulence attributes of *S. mutans* and respective primer sequences used in this study.

Gene	Function	Primer sequence (5′-3′)	
		Forward	Reverse
*gtfB*	Water insoluble glucan Synthesis	AAAGCAACGGATACAGGGGA	CTCTGTCATTGGTGTAGCGC
gtfC	Water soluble and insoluble Glucan synthesis	GGTTTAACGTCAAAATTAGCTGTATTAGC	CTCAACCAACCGCCACTGTT
gtfD	Water soluble glucan synthesis	GAAGTATGGCGGTGCTTTCC	ATAACCAACACCACGGCCTA
vicR	Two-component Regulatory system	TGACACGATTACAGCCTTTGATG	CGTCTAGTTCTGGTAACATTAAGTCCAATA
smu0630	Hypothetical protein involved in biofilm formation	GTTAGTTCTGGTTTTGACCGCAAT	CCCTCAACAACAACATCAAAGGT
comDE	Competence stimulating peptide	ACAATTCCTTGAGTTCCATCCAAG	TGGTCTGCTGCCTGTTGC
16S rRNA	House-keeping gene	ACTCCTACGGGAGGCAGCAG	ATTACCGCGGCTGCTGG

### Impact of UA on acidogenicity

Effect of UA on acid production of *S. mutans* through glycolysis was evaluated by glycolytic pH drop assay and acidity measurement. For glycolytic pH drop assay, *S. mutans* cells was harvested at mid-logarithmic phase, washed in PBS and resuspended in a salt solution containing 50 mM potassium chloride and 1 mM magnesium chloride in the absence and presence of various concentrations of UA (1.5, 2, 2.5, 5, 10 µg mL^−1^). Glucose at 1% w/v final concentration was added and the initial pH of the mixtures was adjusted to pH 7.2–7.4 with 0.2 M potassium hydroxide. Alteration in the pH level by glycolytic activity of *S. mutans* and the impact of UA on the acidogenicity was monitored at 15 min intervals over a period of 120 min^51^.

#### pH of the spent medium

For acidity measurement, THYES broth was prepared with the pH of 7.2. In a sterile 24 well MTP, 1% overnight culture of *S. mutans* was added to 1 mL of THYES broth in the absence and presence of UA at different concentrations (0.25, 0.5, 1, 1.5, 2, 2.5, 5 µg mL^-1^). pH was measured immediately after addition of UA and after 24 h of incubation.

### Effect of UA on acidurity

Role of UA on acid tolerance of *S. mutans* was assessed by measuring the viable count of bacteria after exposure to moderately acidic and extremely acidic pH. *S. mutans* cells cultivated in the absence and presence of various concentrations of UA (1, 1.5, 2 and 2.5 µg mL^−1^) was pelleted by centrifugation at the mid-logarithmic phase. Pellet was equally split into two aliquots namely unadapted and adapted cells. Unadapted cells were directly resuspended into THYES broth of detrimental pH 3.5 and incubated at 37 °C for 2 h. Adapted cells were initially allowed to habituate in the moderately acidic THYES broth of pH 5.5 for 1 h at 37 °C and later exposed to THYES broth of lethal pH 3.5 and further incubated for 2 h at 37 °C. At the end of incubation period, viability of *S. mutans* cell from adapted and unadapted groups were assessed by plating on THB agar plates (pH – 7.5) for 48 h at 37°C^52^.

### H_2_O_2_ sensitivity assay

*S. mutans* culture grown in the absence and presence of various concentrations of UA was adjusted to OD 0.5 at 600 nm. Cells were then swabbed on THA plates and a sterile filter paper disk of 10 mm diameter was placed at the center of plate. Subsequently, the disk was loaded with 15 µL of 30% H_2_O_2_ and incubated for 24 h at 37 °C. After incubation, plates were documented and the zone of clearance was recorded^53^.

Additionally *S. mutans* cells grown to ~ 0.3 OD_600_ nm was exposed to 50 mM H_2_O_2_ in the absence and presence of UA (5 and 10 µg mL^−1^) for 1 h at 37 °C. After incubation, cells were centrifuged, resuspended in PBS and serially diluted. Dilutions were spread plated on THA and incubated at 37 °C for 48 h^54^.

### Influence of UA on eDNA content in *S. mutans* biofilm conglomerate

eDNA content in the biofilm mixture of *S. mutans* was qualitatively analysed by agarose gel electrophoresis. Briefly, *S. mutans* was allowed to form biofilm in the absence and presence of sub-MIC concentrations of UA (0.25, 0.5, 1.0, 1.5 and 2.5 µg mL^−1^) in a 6 well polystyrene plate for 24 h at 37 °C. Subsequently, the planktonic part was removed and loosely bound cells were eliminated by gentle rinse with PBS thrice. Further, the entire biofilm components were scraped from the wells, resuspended in TE buffer (10 mM Tris and 1 mM EDTA, pH 8.0) and vigorously vortexed for 30 min to extricate the complex mixture. The tubes were then centrifuged to collect eDNA comprising supernatant. Eventually, amount of eDNA present in the supernatant was analysed through performing agarose gel electrophoresis (1.5% w/v) followed by ethidium bromide staining^55^.

### Molecular docking analysis

Crystal structures of *S. mutans* response regulator VicR domain (PDB ID: 5ZA3) and GtfC (PDB ID: 3AIC) was obtained from the PDB database. Chemical structure of usnic acid was retrieved from PubChem database (PubChem CID: 5646). Protein was prepared using the AutoDock Tools Version 1.5.6 and the molecular docking was performed through AutoDock Vina software^56^. Protein–ligand interactions were analysed and structural figures were prepared using Maestro 10 (Schrödinger) software.

### Spontaneous resistance development

Development of spontaneous resistance to UA by *S. mutans* was determined as described by Min et al., 2017 with slight modifications^57^. Briefly, overnight cultures of *S. mutans* was washed and the cell culture was adjusted to 1 × 10^8^ CFU/mL in THYES broth which was subsequently confronted with various concentrations of UA on THA plates and incubated for 72 h at 37 °C. After incubation period, number of colonies in control and UA challenged plates were observed and documented using Gel documentation system (Bio-Rad Laboratories, XR+, USA).

### Mature biofilm disruption

To evaluate the effect of biofilm disruptive potency of UA, *S. mutans* was allowed to form mature biofilm. After 24 h, the planktonic part was removed and added with 1 mL of fresh medium together with various concentrations of UA and further incubated for 18 h. At the end of incubation period, cells from control and UA treatment were serially diluted and plated on THA plates which were incubated at 37 °C for 48 h. Plates were documented and variations in viability was noted in CFU/mL. Rest of the cell content from the assay was subjected to FTIR analysis. Briefly, the cells were pelleted down by centrifugation, dried completely under vacuum for 3 h at 50 °C (CHRIST-alpha 2–4 LD plus). Samples were mixed with potassium bromide (KBr) and the spectral scan was taken from 4000 to 400 cm^−1^ with spectral resolution of 4 cm^−1^ (NicoletTM iS5, Thermo Scientific, U.S.A with OMNIC Software).

### Assessment of toxicity on human buccal epithelial cells (HBECs)

As the end application of this study is related to use in dentifrice, the safety evaluation of the compound was assessed with HBECs for which healthy individuals with proper oral hygiene was selected. To collect HBECs, individuals were asked to gently rub on their mucosal surface of cheeks with sterile cotton swab. Sequentially, the swabs were suspended in sterile phosphate buffered saline (PBS) which was used immediately. The suspended cells were pooled and centrifuged at 3000 rpm for 5 min and the pellet was washed thrice in sterile PBS to get rid of debris. A suspension with 5.0 × 10^5^ cells mL^−1^ was prepared by counting the pooled cell suspension through Automated cell counter Countess II FL, Invitrogen, United States). Further, HBECs were incubated with various concentrations of UA (1.0, 1.5, 2.0, 2.5, 5.0 and 10.0 µg mL^−1^) for 20 min at 37 °C to assess detrimental effects, if any. Hydrogen peroxide was included as positive control. At the end of incubation period, cells were stained with 0.1% crystal violet and visualized under microscope (Nikon Eclipse Ts2R, Japan) to assess morphology of the control and UA treated HBECs^58^.

### Impact of UA on oral commensals

Effect of UA on salivary bacteria was assessed through the procedure described by Concannon et al., 2003^59^ with slight modifications^.^ Unstimulated saliva was collected from fourth volunteers with good oral health. Individuals were refrained from eating before 2 h from saliva collection. Saliva was pooled together and subjected to low speed centrifugation to eliminate epithelial cells. Supernatant was centrifuged at 4000 rpm for 15 min at 4 °C to collect saliva bacteria. Pellet containing the salivary commensal was washed thrice with sterile PBS and finally resuspended in the same. 250 µL of the bacterial suspension was added with UA at a final concentration of 10 µg mL^−1^. Appropriate vehicle control was maintained in parallel. Bacterial suspension exposed to 0.12% chlorhexidine was considered as positive control. After a brief exposure of 10 min (to mimic the exposure time of the oral care products) at 37 °C, the compounds were removed by three rounds of centrifugation. Finally, bacterial pellet suspended in the PBS was serially diluted and spread platted on Muller Hinton agar plates.

### Statistical analysis

All the experiments were carried out in at least three biological replicates with at least two technical replicates and values are presented as Mean ± standard deviation (SD). To analyse the significant difference between the value of control and treated samples, one-way analysis of variance (ANOVA) and Duncan’s post hoc test was performed with the significant *p* value of < 0.05 by the SPSS statistical software package version 17.0 (Chicago, IL, United States).

## Supplementary information

Supplementary information.
