# Supplementary material for: Usnic acid deteriorates acidogenicity, acidurance and glucose metabolism of Streptococcus mutans through downregulation of two-component signal transduction systems
Source: Sci Rep. 2021 Jan 14;11:1374. doi: 10.1038/s41598-020-80338-6 (PMC7809355; doi:10.1038/s41598-020-80338-6)
Supplement: Supplementary file 1 — Supplementary information. [file 41598_2020_80338_MOESM1_ESM.pdf]

Usnic acid deteriorates acidogenicity, acidurance and glucose metabolism of *Streptococcus mutans* through downregulation of Two-component signal transduction systems

Arumugam Priya, Chandra Bose Manish Kumar, Alaguvel Valliammai, Anthonymuthu Selvaraj and Shunmugiah Karutha Pandian*

Department of Biotechnology, Alagappa University, Science Campus,

Karaikudi 630003, Tamil Nadu, India.

*Address for correspondence: Shunmugiah Karutha Pandian: pandiansk@gmail.com; Fax: +91 4565 225202; Tel: +91 4565 225215

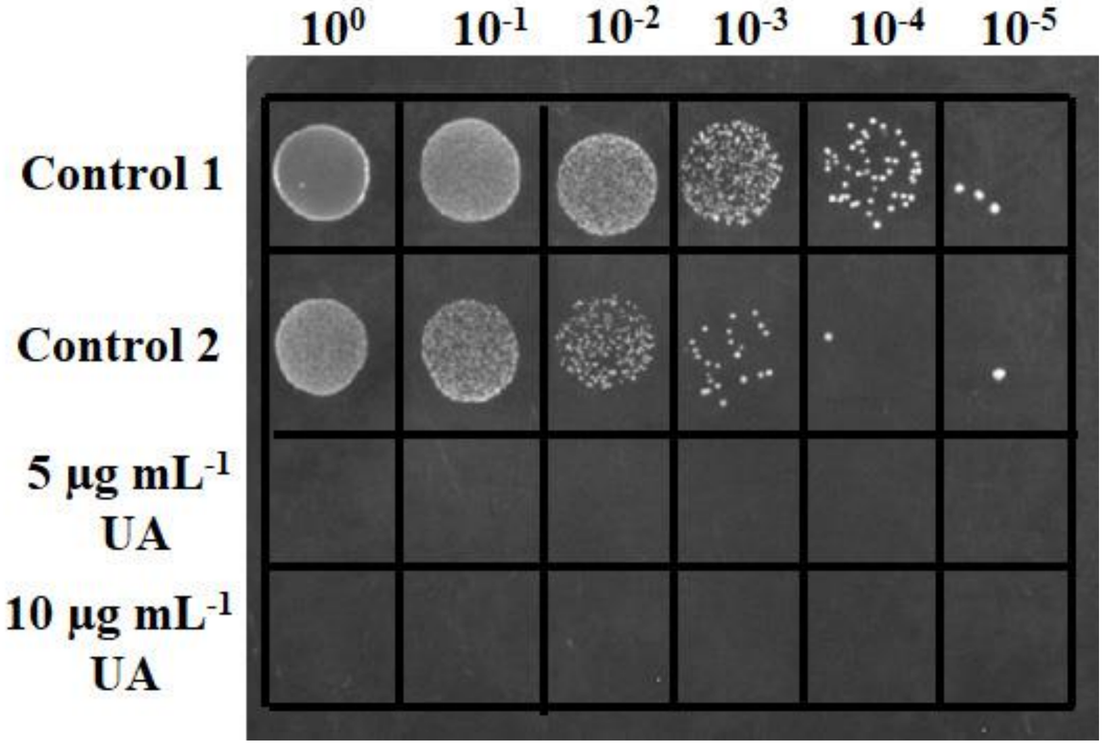

Supplementary Figure S1: Spot assay confirming the increased sensitivity of *S. mutans* cells treated with UA to H_2O_2 treatment. Control – 1 *S. mutans* cells unexposed to H_2O_2 treatment; Control – 2 *S. mutans* cells exposed to 50 mM H_2O_2. Complete killing of *S. mutans* cells were observed with the simultaneous exposure of H_2O_2 and UA.

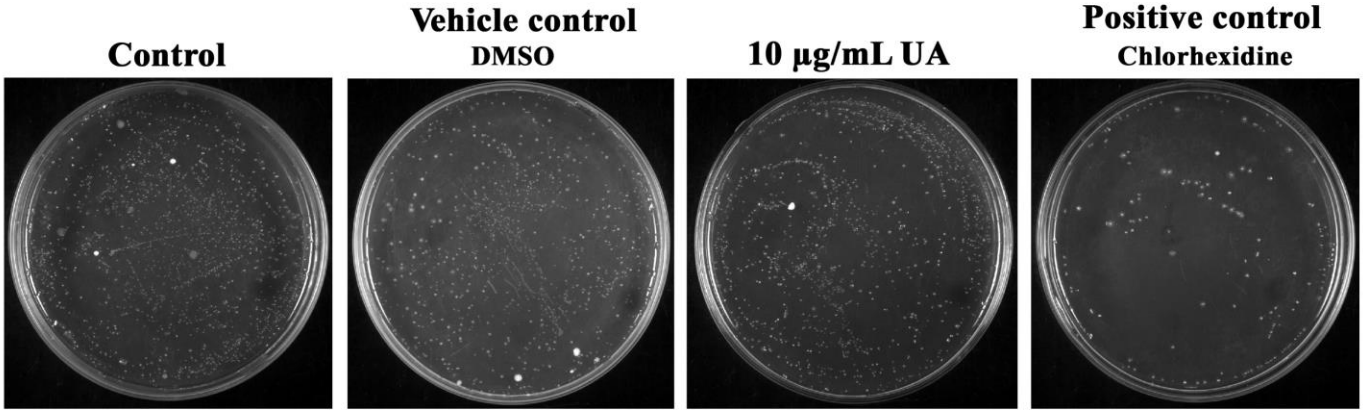

Supplementary Figure S2: Effect of UA on oral commensals. Short term exposure of UA to salivary bacteria did not show a significant change in the oral commensals substantiating the therapeutic potential of UA in oral care products.
